# Impact of nitrogen compounds on fungal and bacterial contributions to codenitrification in a pasture soil

**DOI:** 10.1038/s41598-019-49989-y

**Published:** 2019-09-16

**Authors:** David Rex, Timothy J. Clough, Karl G. Richards, Leo M. Condron, Cecile A. M. de Klein, Sergio E. Morales, Gary J. Lanigan

**Affiliations:** 1 Department of Soil and Physical Sciences, Lincoln University, Lincoln, New Zealand; 2 Teagasc, Environmental Research Centre, Johnstown Castle, Wexford, Ireland; 3 AgResearch Invermay, Mosgiel, New Zealand; 4 Department of Microbiology and Immunology, School of Biomedical Sciences, University of Otago, Dunedin, New Zealand

**Keywords:** Grassland ecology, Stable isotope analysis, Microbial ecology, Element cycles, Climate-change mitigation

## Abstract

Ruminant urine patches on grazed grassland are a significant source of agricultural nitrous oxide (N_2_O) emissions. Of the many biotic and abiotic N_2_O production mechanisms initiated following urine-urea deposition, codenitrification resulting in the formation of hybrid N_2_O, is one of the least understood. Codenitrification forms hybrid N_2_O via biotic N-nitrosation, co-metabolising organic and inorganic N compounds (N substrates) to produce N_2_O. The objective of this study was to assess the relative significance of different N substrates on codenitrification and to determine the contributions of fungi and bacteria to codenitrification. ^15^N-labelled ammonium, hydroxylamine (NH_2_OH) and two amino acids (phenylalanine or glycine) were applied, separately, to sieved soil mesocosms eight days after a simulated urine event, in the absence or presence of bacterial and fungal inhibitors. Soil chemical variables and N_2_O fluxes were monitored and the codenitrified N_2_O fluxes determined. Fungal inhibition decreased N_2_O fluxes by ca. 40% for both amino acid treatments, while bacterial inhibition only decreased the N_2_O flux of the glycine treatment, by 14%. Hydroxylamine (NH_2_OH) generated the highest N_2_O fluxes which declined with either fungal or bacterial inhibition alone, while combined inhibition resulted in a 60% decrease in the N_2_O flux. All the N substrates examined participated to some extent in codenitrification. Trends for codenitrification under the NH_2_OH substrate treatment followed those of total N_2_O fluxes (85.7% of total N_2_O flux). Codenitrification fluxes under non-NH_2_OH substrate treatments (0.7–1.2% of total N_2_O flux) were two orders of magnitude lower, and significant decreases in these treatments only occurred with fungal inhibition in the amino acid substrate treatments. These results demonstrate that *in situ* studies are required to better understand the dynamics of codenitrification substrates in grazed pasture soils and the associated role that fungi have with respect to codenitrification.

## Introduction

The nitrous oxide (N_2_O) molecule is a potent greenhouse gas, with a global warming potential 298 times that of carbon dioxide over a 100 year time period^1^. It is also a precursor to reactions involved in the depletion of stratospheric ozone^2^. A major source of anthropogenic N_2_O emissions is the intensive grazing of grasslands and the resulting ruminant urine deposition that occurs^3,4^. Thus, in order to achieve mitigation of N_2_O emissions from intensively managed pasture soils it is important to identify and understand the processes that lead to N_2_O formation and consumption within ruminant urine-affected soil.

Typically, ruminant urine-N deposited onto pasture soil is comprised of >70% urea-N. Upon contact with the soil the urea begins to hydrolyse, forming ammonium (NH_4_^+^) resulting in a rapid elevation of soil pH to 8.0 or higher^5^. The equilibrium between NH_4_^+^ and ammonia (NH_3_) is pH driven^6,7^. Soil pH >7.0 leads to elevated NH_3_ concentrations in the soil, that not only result in NH_3_ volatilization^8^ but which can also inhibit the microbial oxidation of nitrite (NO_2_^−^) by *Nitrobacter sp*.^9,10^. As the pH decreases to ca. <7.0, the equilibrium between NH_4_^+^ and NH_3_ shifts in favour of NH_4_^+^, which may undergo clay mineral fixation, plant uptake, immobilization or nitrification^11^.

Production of N_2_O may occur via the microbial pathways of nitrification, denitrification, and nitrifier-denitrification^12^. However, under ruminant urine-affected soil it is bacteria, not archaea, that respond to the high concentration of NH_4_^+^ substrate that forms in the soil following ruminant urine deposition^13,14^, since bacterial nitrifiers operate under conditions of high inorganic NH_4_^+^ inputs^14–16^. During the conventional nitrification process bacteria produce N_2_O as a by-product of NH_2_OH oxidation^17^ or during nitrifier-denitrification following nitric oxide (NO) reduction^15^. However, the major source of N_2_O emissions from ruminant urine-affected soil occurs as a result of the NO_3_^−^ formed, as a consequence of nitrification. Under anaerobic conditions microbes denitrify NO_3_^−^ to sequentially form NO_2_^−^, NO and N_2_O, which are all obligate intermediaries of the denitrification pathway^12,18–20^ to finally create dinitrogen (N_2_). In order to conserve both energy and oxygen, nitrifier-denitrification may occur in response to limited soil oxygen conditions^21^, whereupon nitrifiers convert NO_2_^−^ to NO, N_2_O and N_2_^12^ although the significance of this process may have been overestimated in some studies^22^. In addition to these N_2_O production pathways, N_2_O may also be produced as ‘hybrid’ N_2_O via codenitrification, a process involving two different N pools^20,23^. Spott *et al*.^20^ reviewed possible biotic and abiotic reactions that may be included under the term ‘codenitrification’. For example, abiotic reactions involving reduced iron (Fe^2+^) and NO_2_^−^, may occur at the interface between an aerobic zone overlying an anaerobic zone when NO_2_^−^ diffusing downwards meets Fe^2+^^24,25^. However, this process is unlikely to contribute significantly to N_2_O emissions due to insufficient Fe^2+^ ion concentrations in most soils^26,27^. A more common abiotic reaction that occurs in acidic soil (pH < 5.0) is that of chemodenitrification (abiotic-nitrosation), whereby NO_2_^−^ and H^+^ react to form nitrous acid (HNO_2_), which can then react with amino compounds, NH_2_OH, NH_4_^+^ or other organic N compounds resulting in the formation of N_2_O^28,29^. However, under alkaline conditions when oxygen is depleted codenitrification may occur via biologically mediated nitrosation^20,30^. Under such conditions the hydrogen atom in an organic compound is replaced with a nitroso group (−N=O). Enzymatic nitrosyl compounds attract nucleophile compounds (e.g. NH_2_OH, NH_4_^+^, hydrazine (N_2_H_2_), amino compounds and NH_3_) resulting in hybrid N_2_O or N_2_ species, containing one N atom derived from the nucleophile and one N atom derived from the nitrosyl compound^20^. Recent studies have revealed the significant contribution of codenitrification to gaseous N losses from grassland soils^30–32^. Using a ^15^N tracer approach, Laughlin and Stevens^32^ found evidence for fungal dominated ^15^NO_3_^−^ depletion leading to hybrid N_2_ emissions where 92% of the N_2_ evolved was derived from codenitrification. Selbie *et al*.^30^ confirmed, *in-situ*, the dominance of codenitrification derived N_2_ under urine patch conditions when 56% of applied urine was codenitrified. Recently, studies have found further evidence for N_2_O production via codenitrification under simulated ruminant urine patch conditions^31,33^. However, knowledge about the nucleophile species that potentially partake in codenitrification under ruminant urine patch conditions is still lacking. Different N substrates (as potential nucleophiles) such as amino acids, NH_4_^+^ and NH_2_OH have previously been proven to be capable of generating hybrid N_2_O/N_2_ *in vitro* when utilized by one microbial species in combination with either NO_3_^−^ or NO_2_^−^^34–37^. Amino acids have been reported to be freely available within the soil solution, for example, phenylalanine (8–50 µg N g^−1^ soil) and glycine (35–193 µg N g^−1^ soil) were measured in long-term agricultural land on a Stagni-Haplic Luvisol^38^ and in different cattle manure treated crop fields on a sandy Orthic Luvisol^39^. Reported concentrations of NH_2_OH are orders of magnitude lower, for example, Liu *et al*.^40^ reported concentrations of <0.0348 µg N g^−1^ in a forest soil, while NH_4_^+^ and NH_3_ are routinely reported following ruminant urine deposition events^41^. Therefore, we hypothesise that in a soil matrix under simulated ruminant urine deposition the N substrates applied in this study will be utilized for codenitrification reactions, with a microbial preference for NH_2_OH and that these reactions would be mainly fungi driven.

## Results

### Soil pH, and mineral N

**Figure 1:**
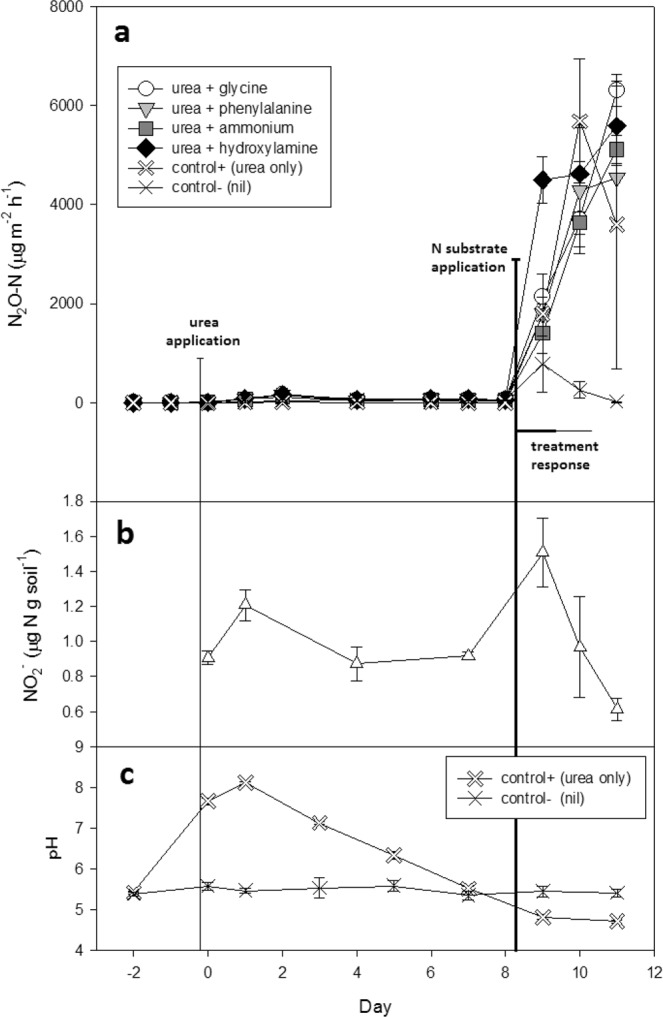
Soil response to urea and treatment application. The N_2_O fluxes over time (**a**) of the no inhibition treatments. Before Day 9 the N_2_O fluxes did not significantly differ between the positive control and the treatments. On Day 9, the N_2_O fluxes of all treatments and the positive control increased as listed in Table 1, for simplicity only the non-inhibition treatments are depicted in Fig. 1 to visualize the range of increase. Below the NO_2_^−^ concentration in the soils as measured in the NO_2_^−^ control. (**b**) These partially destructive analysis was not performed within the treatment soils and the positive controls, but depicts the assumed NO_2_^−^ concentration development within these soils. The soil surface pH was measured in all jars, however, all treatment soil surface pH values did not differ from the depicted positive control, in contrast to the negative control. (**c**) Each symbol represents a mean of n = 3, all error bars are ± SD.

Within 6 h of applying the urea solution to the soil surface pH values increased uniformly in all treatments from an average of 5.6 ± 0.2 on Day −2 to >7.6 on Day 0. The surface soil pH peaked 30 h after the urea application, at 7.9, followed by a steady decline to 4.8 ± 0.1 on Day 9 (Fig. 1) in the positive control and all treatments. The surface pH in the negative control ranged from 5.4 ± 0.05 to 5.6 ± 0.06 over the course of the experiment (Fig. 1).

Soil NO_2_^−^ concentrations were significantly elevated within the first 4 days following urea application (p < 0.05). Soil NO_2_^−^ concentrations peaked at 1.5 ± 0.2 µg NO_2_^−^-N g^−1^ soil on Day 9, subsequent to the physical mixing and then decreased to 0.6 ± 0.1 µg NO_2_^−^-N g^−1^ soil on Day 11 (Fig. 1b).

Both the soil NO_3_^−^ and NH_4_^+^ concentrations were higher (p < 0.01) in the positive control at Day 12 compared with the negative control. The NO_3_^−^ concentrations in the positive control were in the range of 366 ± 122 µg NO_3_^−^-N g^−1^ soil while NH_4_^+^ concentrations were 174 ± 7 µg NH_4_^+^-N g^−1^ soil. The soil NO_3_^−^ and NH_4_^+^ concentrations in the negative control were 64 ± 23 µg NO_3_^−^-N g^−1^ soil and 22 ± 1 µg NH_4_^+^-N g^−1^ soil, respectively.

### N_2_O fluxes

Initially N_2_O fluxes increased within the first 48 h following urea application, with treatments and positive controls emitting 100–200 µg N_2_O-N m^−2^ h^−1^. From Day 4 to Day 8, the N_2_O fluxes from the urea-treated soil were <100 µg N_2_O-N m^−2^ h^−1^ across all treatments. Following N_2_O flux measurement on Day 8, the process of mixing the soil and/or the addition of N substrates increased N_2_O fluxes at Day 9 (Fig. 1). In the absence of microbial inhibition, the addition of the NH_2_OH substrate resulted in higher N_2_O fluxes (4496 µg N_2_O-N m^−2^ h^−1^) when compared to the amino acid (1796 to 2130 µg N_2_O-N m^−2^ h^−1^) and NH_4_^+^ (1405 µg N_2_O-N m^−2^ h^−1^) treatments on Day 9 (p < 0.001), 24 h after N substrate addition.

The magnitude of the decrease in the N_2_O fluxes, following inhibition treatment, varied due to inhibitor type and N substrate applied (Table 1). The N_2_O emissions were lower under fungal inhibition by 46, 34 and 21% in the glycine, phenylalanine, and NH_2_OH treatments, respectively, while fungal inhibition did not affect fluxes from the NH_4_^+^ treatment. Bacterial inhibition decreased N_2_O fluxes by 14, and 26% in the glycine and NH_2_OH treatments, respectively, while fluxes from the phenylalanine and NH_4_^+^ treatments were unaffected by bacterial inhibition (Table 1). Applying both inhibitors simultaneously (combined inhibition) resulted in N_2_O fluxes decreasing by 29–41% in all N substrate treatments (Table 1). In the glycine treatment fungal inhibition decreased N_2_O fluxes more than bacterial inhibition, but this decrease was not enhanced when the two inhibitors were combined (Table 1). While bacterial inhibition did not significantly lower N_2_O fluxes in the phenylalanine treatment, the fungal inhibition either alone or within the combined inhibition did decrease N_2_O fluxes (Table 1). Sterilizing effectively eliminated N_2_O fluxes in both the amino acid treatments, and the NH_4_^+^ treatment (Table 1). However, this was not the case when NH_2_OH was applied, where emissions decreased by 72% (Table 1).

**Table 1:** Emission rates of total N_2_O (µg N_2_O-N m^−2^ h^−1^) of the inhibitor × N substrate treatments on Day 9.

N substrate	no inhibition	fungal inhibition	bacterial inhibition	combined inhibition	sterilized soil	test & significance
Glycine	2130 **a** ± 134	1144 **c** ± 177	1830 **b** ± 163	1331 **c** ± 114	2 **d** ± 0	Holm-Sidak*
Phenyl.	1796 **a** ± 333	1182 **b** ± 66	1705 **a** ± 36	1267 **b** ± 93	1 **c** ± 1	t-tests*
Ammonium	1405 **a** ± 49	1142 **ab** ± 301	1010 **ab** ± 873	904 **b** ± 111	3 **c** ± 0	Tukey**
Hydroxy.	4496 **a** ± 467	3563 **b** ± 358	3324 **bc** ± 240	2671 **c** ± 253	1246 **d** ± 21	Holm-Sidak*

As taken 24 h after the microbial inhibition, these data represent the N_2_O emissions during the overlapping time of N substrates starting to contribute to N_2_O emissions and still working microbial inhibitors. Different statistical analyses have been used to determine differences, dependent on normal or non-normal distributed data and homogeneous or inhomogeneous variances.

Values are means (n = 3) with standard deviation, different letters indicate the level of significance based on the mentioned test, where all inhibition treatments for each N substrate are tested against each other. Level of significance: *p < 0.05, **p = 0.001.

### N_2_O-^15^N enrichment

The positive control (urea only at natural abundance) had a N_2_O-^15^N enrichment of 0.363 ± 0.004 (SD) on Day 9. At the same time, the addition of an N substrate resulted in small increases in the N_2_O-^15^N enrichments in all treatments with the following exceptions (Table 2): the phenylalanine treatment with either no inhibition or bacterial inhibition, and the NH_4_^+^ treatment with bacterial inhibition (Table 2). Within a given N substrate treatment, when comparing the N_2_O-^15^N enrichment of the no inhibition treatment and a specific inhibitor treatment, few treatment differences occurred. Under glycine only the sterilized soil treatment varied, with a higher N_2_O-^15^N enrichment relative to the no inhibition treatment (Table 2). Applying phenylalanine also resulted in enhanced N_2_O-^15^N enrichment, mostly when applied to the sterilized soil but this was not statistically different from the no inhibition treatment (Table 2). With NH_4_^+^ as the N substrate the N_2_O-^15^N enrichment was again highest in the sterilized soil treatment, but none of the inhibitor treatments caused N_2_O-^15^N enrichment to differ from the no inhibitor treatment (Table 2). The biggest shifts in N_2_O-^15^N enrichment with inhibition treatments occurred in the NH_2_OH treatment where applying bacterial inhibition, either alone or within the combined inhibition treatment, caused significant decreases in N_2_O-^15^N enrichment relative to the no inhibition treatment (Table 2).

**Table 2:** N_2_O ^15^N enrichment (atm%) of the inhibitor × N substrate treatments on Day 9, 24 h after the treatment application.

N substrate	no inhibition	fungal inhibition	bacterial inhibition	combined inhibition	sterilized soil	test & significance
Glycine	0.370 **b** ± 0.001	0.380 **ab** ± 0.001	0.373 **ab** ± 0.002	0.375 **ab** ± 0.006	1.211 **a** ± 0.104	Tukey*
Phenyl.	*0*.*363* **ab** ± *0*.*003*	0.377 **ab** ± 0.003	*0*.*360* **b** ± *0*.*003*	0.378 **ab** ± 0.011	0.900 **a** ± 0.170	Tukey*
Ammonium	0.481 **ab** ± 0.034	0.374 **b** ± 0.003	0.475^ǂ^ ± 0.026	0.384 **ab** ± 0.003	0.896 **a** ± 0.088	Tukey*
Hydroxy.	41.587 **a** ± 1.414	43.147 **a** ± 4.055	27.165 **b** ± 1.555	30.384 **b** ± 3.499	44.219 **a** ± 4.625	Dunn’s Method*

Different statistical analyses have been used to determine differences, dependent on normal or non-normal distributed data and homogeneous or inhomogeneous variances.

Values are means (n = 3) with standard deviation, different letters indicate the level of significance (p < 0.05) based on the mentioned test where all inhibition treatments for each N substrate are tested against each other. For phenylalanine the italic font indicates no significant difference if compared to the positive control (+urea, nil N substrate) on the same day (0.363 ± 0.004). ^ǂ^For bacterial inhibition of the ammonium substrate n = 2, thus and it is excluded from the Tukey analysis.

### N_2_O codenitrification

Increased ^15^N enrichment of the N_2_O fluxes revealed the formation of hybrid N_2_O (codenitrified N_2_O (N_2_O_co_)). Amino acid and NH_4_^+^ treatments emitted 13–17 µg N_2_O_co_-N m^−2^ h^−1^ in the case of no inhibition, while bacterial inhibition and/or fungal inhibition lowered these fluxes by >30% (Table 3). With sterilized soil under these N substrate treatments codenitrification fluxes ceased (Table 3). The N_2_O_co_ fluxes from the NH_2_OH treatment decreased significantly in the presence of the combined inhibition (>46%, Table 3) but not when applied individually. Under NH_2_OH, hybrid N_2_O fluxes equalled 3851 µg N_2_O_co_-N m^−2^ h^−1^ with no inhibition present. Sterilizing the soil significantly lowered NH_2_OH derived codenitrification fluxes to 617 µg N_2_O_co_-N m^−2^ h^−1^. This corresponded to a decrease of >83%, compared to the no inhibition treatment; or a decrease of >71%, compared to the combined inhibitor treatment (Table 3).

**Table 3:** Codenitrification fluxes (N_2_O_co_, µg N_2_O-N m^−2^ h^−1^) of the inhibitor × N substrate treatments on Day 9, 24 h after the treatment application.

N substrate	no inhibition	fungal inhibition	bacterial inhibition	combined inhibition	sterilized soil	test & significance
Glycine	16 **a** ± 0	9 **b** ± 0	14 **a** ± 0	10 **b** ± 0	0 **c** ± 0	Holm-Sidak*
Phenyl.	13 **a** ± 0	9 **b** ± 0	12 **ab** ± 0	10 **ab** ± 0	0 **c** ± 0	Holm-Sidak*
Ammonium	17 **a** ± 0	9 **ab** ± 0	12 **ab** ± 4	7 **ab** ± 0	0 **b** ± 0	Tukey*
Hydroxy.	3851 **a** ± 365	3432 **ab** ± 717	3034 **ab** ± 190	2198 **b** ± 853	617 **c** ± 138	Holm-Sidak*

Different statistical analyses have been used to determine differences, dependent on normal or non-normal distributed data and homogeneous or inhomogeneous variances.

Values are means (n = 3) with standard deviation, different letters indicate the level of significance based on the mentioned test where all inhibition treatments for each N substrate are tested against each other. Level of significance: *p < 0.05.

## Discussion

The hydrolysis of urea and its resulting products increases NH_4_^+^ and OH^−^ concentrations in the soil^5^ with the latter responsible for the elevated soil surface pH observed in treatments containing urea. Urea application elevated soil NH_4_^+^-N concentrations, as evidenced by the higher concentrations in the positive control when compared with the negative control. Elevated soil pH will have resulted in the NH_4_^+^/NH_3_ equilibrium shifting towards NH_3_^5^_._ However, by Day 8 the concentration of NH_3_ will have been relatively low based on soil pH values at this time^5^. While NH_3_ can inhibit NO_2_^−^ oxidisers under urea-affected soil^9,10^ the elevated soil NO_3_^−^-N concentrations at the end of the experiment and the decline in NO_2_^−^ from Day 1 to 7 demonstrates NO_2_^−^ oxidisers were functioning. The soil NO_3_^−^-N concentration on Day 9 was higher when compared to a previous study by Rex *et al*.^33^, at a similar time following urea application. This higher soil NO_3_^−^-N concentration is likely to have occurred due to the reduced potential for nitrifier inhibition^9,10^, a consequence of the lower urea-N rate used in the current study. Considering the soil pH and inorganic-N dynamics it can be concluded that the application of urea was representative of conditions under a typical urine patch^41,42^, and that the N substrate treatments were applied during a period of relatively rapid inorganic-N transformation.

The rapid increase in N_2_O fluxes following inhibitor application was partially the result of physically mixing the soil in order to distribute the inhibitors, which resulted in entrapped N_2_O, in the soil, being released^43^. Furthermore, soil, not previously exposed to oxygen, would have become exposed and thus there is also the possibility that inhibition of N_2_O reductase occurred, preventing complete denitrification^44^. However, the application of substrate-N also contributed to the N_2_O flux as demonstrated by the increased N_2_O-^15^N enrichments, particularly in the case of the NH_2_OH treatment (Fig. 1a).

Soil N_2_O emissions are strongly driven by the presence and turn-over of NO_2_^−^ which is the ‘gate-way molecule’ for N_2_O production^9,45^. In the current study soil NO_2_^−^ concentrations were elevated on Day 9 but at concentrations lower than previously observed (e.g. Clough *et al*.^31^) due to the lower urea application rate in the current study preventing NH_3_ inhibition of NO_2_^−^ oxidation^45^. Hence, the ensuing N_2_O emissions most likely result from the net effects of microbial processes utilising NO_2_^−^ and/or the N substrate added.

The effects of the microbial inhibitors, cycloheximide, streptomycin and heat sterilization on N_2_O production were assessed 12 h after inhibitor application since maximum efficacy is reported within 24 h of application^46^. The decline in the N_2_O fluxes following fungal inhibition within the amino acid and NH_2_OH treatments demonstrates fungal mechanisms were responsible for a portion of the N_2_O produced (21–46%). Previous studies have shown fungi are able to produce N_2_O^32,33,47,48^. Nitric oxide reductase (P450nor), is a key feature of fungal denitrification and has been observed to require hypoxia and either NO_3_^−^ or NO_2_^−^ substrate to generate N_2_O^47,49^: these conditions occurred within the current study. Biotic N_2_O emissions from non-autoclaved soil suspensions can be stimulated by the presence of both NH_2_OH and NO_3_^−^, as was the case in the NH_2_OH treatment of the current study. Thus, the decline in N_2_O emissions in the NH_2_OH treatment, with fungal inhibition, implies a fungal mechanism was partially responsible for the N_2_O flux, via NH_2_OH utilisation.

With bacterial inhibition, the decline in the N_2_O flux under the NH_2_OH treatment likely occurred due to the bacterial inhibitor preventing the function of the ammonia oxidising bacteria (AOB), which utilise NH_2_OH to gain energy^50^. Increased mRNA transcription levels of the functional genes present in AOB that encode for NH_2_OH oxidoreductase (*haoA*), and the reductases for NO_2_^−^ and NO, which are *nirK* and *norB*, respectively, become elevated following NH_2_OH application^50^. A similar result and explanation might have been expected following bacterial inhibition in the NH_4_^+^ treatment, given that NH_2_OH is an intermediate in the nitrification pathway, however the result was not statistically significant (Table 1). Lower N_2_O fluxes from the glycine treatment under bacterial inhibition may have also resulted from a diminished nitrification rate of the NH_4_^+^ derived from the mineralized glycine-N, and thus delivering less NO_2_^−^ to the soil pool. However, this did not occur under the phenylalanine treatment possibly because it is a more complex molecule and potentially slower to be mineralized, and thus potentially bacteria played less of a role in the N_2_O fluxes derived from phenylalanine. Again, with glycine the combined inhibition treatment demonstrated the role of fungi in generating N_2_O. This was also the case with phenylalanine where the combined inhibition cut N_2_O emissions to a level comparable to fungal inhibition alone.

The near complete suppression of N_2_O emissions in the amino acid and NH_4_^+^ treatments, under the combined inhibition treatment, demonstrates that the observed N_2_O fluxes were almost entirely from biologically driven processes. As previously shown, from the δ^13^C signatures of respired amino acid–CO_2_-C, amino acids are readily mineralized, forming NH_4_^+^^51^. Consequently, amino acids will contribute to N_2_O fluxes if this NH_4_^+^ is nitrified, or via the denitrification of the nitrification products^51^. The residence time of amino acids in soils is generally reported in hours and depends on soil type^51–53^. However, the lack of a significant N_2_O flux response to amino acid and NH_4_^+^ substrate additions at Day 9, relative to the positive control (Fig. 1), is most likely due to the large background NH_4_^+^ pool present at the time of N substrate addition, derived from the urea addition. Hence, the NH_4_^+^ formed from either amino acid mineralization or direct NH_4_^+^ addition will have been diluted by at least 10-fold, assuming all substrate-N was immediately available. Furthermore, it is likely other amino acids were also present to further dilute the amino acid additions. For example, after extracting three soils McLain and Martens^51^ found the sum of 18 amino acids to range from 9 to 20 g kg^−1^ of soil, when examining an arid grassland (Well-drained Typic Torrifluvents of the Pima series). In contrast to the soil used in this study, these amino acid concentrations referred to a non-irrigated soil with an expected lower microbial abundance.

With the exception of NH_2_OH, the near-zero N_2_O emissions after applying the N substrates to the sterilized soils indicated that the N_2_O fluxes were dominated by biotic processes. This was not the case for NH_2_OH where the N_2_O flux from the sterilized soil was ∼28% that of the no inhibition treatment. It has previously been shown that the NH_2_OH molecule may decompose abiotically to produce N_2_O^50,54–56^.

The lack of any corresponding shifts in the relatively low ^15^N enrichments of the N_2_O evolved from the amino acid treatments, under the various inhibition treatments, suggests fungi were not directly utilising the amino acids for N_2_O production. The codenitrification product depends on the redox state of the N-donor, and prior studies have shown amines (-R-NH_2_) to be codenitrified to N_2_^47^. Thus, the lack of any corresponding shifts in the relatively low ^15^N enrichments of the N_2_O evolved from the amino acid treatments may have also been the result of N_2_ being produced. Despite this, fungal inhibition lowered amino acid derived codenitrified N_2_O (Table 3), indicating that products derived from the amino acid mineralization are involved in fungal codenitrification. The lack of any bacterial inhibition effect on the codenitrification flux demonstrates the dominant role of fungi in codenitrification^33^.

Increasing ^15^N enrichment of the N_2_O molecule demonstrates that the N_2_O-N partially derives from a ^15^N enriched source. In the case of the NH_2_OH, applied with an enrichment of 98 atom% ^15^N, the highly ^15^N enriched N_2_O emissions demonstrate the applied NH_2_OH contributed strongly to the evolved N_2_O flux.

Using soil suspensions Spott and Stange^57^ concluded N_2_O production from NH_2_OH in soil was complex due to the interaction of production pathways involving both abiotic formation and biogenic formation, resulting from both codenitrification and denitrification. Adding the NH_2_OH substrate to the sterilized soil (abiotic conditions) the ^15^N enrichment of the N_2_O (∼44 atom%) aligned closely with the calculated ^15^N enrichment of 49 atom% that indicates hybrid N_2_O production via abiotic N-nitrosation. The formation of N_2_O via NH_2_OH reacting with NO_2_^−^ occurs due to abiotic nitrosation processes^58^, and has been previously observed in sterilized soils^56^. The NH_2_OH compound has also been reported to decay abiotically to form N_2_O with the process slowed down when NO_2_^−^ is preesent^58^. However, had this been the main process for N_2_O formation the ^15^N enrichment of the N_2_O evolved would have aligned more with the applied NH_2_OH-^15^N enrichment. The combined inhibition treatment significantly decreased the N_2_O codenitrification flux by 50% (Table 3) compared to the no inhibition treatment (Table 2) indicating abiotic reactions were also contributing substantially to the observed N_2_O flux.

Fungi contributed to N_2_O production when NH_2_OH was applied, as indicated by the flux decrease under the fungal inhibition treatment, however, the lack of any change in the N_2_O-^15^N enrichment indicates fungal inhibition was not affecting the process generating ^15^N enriched N_2_O. Conversely, the further decrease in both the N_2_O flux and N_2_O-^15^N enrichment in the bacterial inhibition and the combined inhibition treatments, showed that the N_2_O production process was inhibited, and that less ^15^N enriched NH_2_OH contributed to the N_2_O flux produced. Therefore, the codenitrification flux also tended to decline in the presence of the bacterial inhibitor. Bacterial inhibition diminishes, amongst others, the activity of AOB and thus (i) lowers the consumption of NH_2_OH via bacterial nitrification, (ii) lowers the enrichment of the nitrification products derived from ^15^N enriched NH_2_OH, and thus (iii) the formation of ^15^N enriched nitrification intermediaries NO_2_^−^ and NO declines. Since NO_2_^−^ and NO have been shown be involved in codenitrification, decreases in the concentration of these molecules would lead to lower N_2_O fluxes with lower ^15^N enrichment. Furthermore, had ^15^N enriched NH_2_OH progressed to NO_2_^−^ then any denitrification of this NO_2_^−^ that contributed to the ^15^N enriched N_2_O pool, would also have occurred at a slower rate or been prevented with inhibition of bacterial denitrifiers.

## Conclusions

Codenitrification occurs when N-donors, such as those studied here (NH_4_^+^, glycine, phenylalanine and NH_2_OH) react with a nitrosyl compound, to form hybrid N_2_O. Using selective microbial inhibition treatments, and simulating a ruminant urine patch environment, we demonstrated that all the used ^15^N-labelled N substrates contributed to codenitrification in a soil matrix. Hydroxylamine was the most important N substrate with respect to increasing the N_2_O flux and contributing to codenitrification (85.7% of total flux), likely because of its more reactive character compared to the other N substrates. The codenitrification N_2_O fluxes following amino acid-^15^N addition were orders of magnitude lower (0.7–1.2% of total flux), potentially due to dilution from antecedent amino acids or their break down products, which in turn means that a contribution of these natural amino acids could be assumed under the experimental conditions. Fungal inhibition resulted in a significant decline in the formation of amino acid derived codenitrification fluxes, underlining once more the importance of fungal codenitrification vs. bacterial codenitrification. The relatively lower codenitrification N_2_O fluxes with amino acids may also be a result of the microbial community structure that is present^20^. Alternatively, codenitrification of NH_2_OH to form N_2_O has been reported in the absence of organic electron donors^59^ hence, given that codenitrification is in principle dependent on organic carbon respiration a lack of organic substrate or variations in its form may have favoured codenitrification of NH_2_OH^20^. The results of this study, demonstrated that codenitrification occurs via multiple pathways in a pasture soil following a simulated bovine urine event. Codenitrification resulting from the presence of NH_2_OH is likely to be the dominant process, in the short-term following the deposition of ruminant urine with its relatively high urea-N loading. The results warrant further *in situ* investigation of the dynamics of potential N-donors, in conjunction with N_2_O fluxes, under ruminant urine patches.

## Materials and Methods

### Experimental design

A bulked soil sample was taken from a sandy loam pasture soil on the Lincoln University dairy farm (0–10 cm), New Zealand (43°38′25.23″S, 172°27′24.71″E, Typic Immature Pallic Soil, (USDA: Udic Haplustept)). The pasture consisted of perennial rye grass (*Lolium perenne L*.) and white clover (*Trifolium repens L*.). Field moist soil was sieved (4 mm) to remove stones and plants and then placed into jars (250 mL, Ø 8.1 cm), corresponding to 100 g dry weight (ca. 82 cm^3^), and moistened to 50% of water-holding capacity^33^ (ca. 83% water-filled pore space).

Initially the jars, with soil, were placed in an incubator, in the dark, at 23 °C and wetted-up daily to preincubation weight. After four days, any germinated weed seedlings were removed and the experimental period of 14 days commenced (Day −2 to Day 11). An aqueous urea solution (500 µg urea-N g dry soil^−1^) was applied on Day 0 in order to simulate a bovine urine deposition event^31,60^. On Day 8, microbial inhibition treatments were applied with the N substrate treatments applied immediately after this in an aqueous solution (4 mL) as noted below.

Treatments consisted of ^15^N enriched N substrate species (glycine (98), L-phenylalanine (98), NH_4_^+^ (99) and NH_2_OH (98); atom% ^15^N enrichment in bracket) with each N substrate treatment further split into five microbial inhibition treatments (no inhibition, fungal inhibition, bacterial inhibition, fungal and bacterial inhibition (‘combined inhibition’) and soil total microbial inhibition (heat sterilised soil)). Treatments were replicated thrice. The amino acid-N concentrations were based on the findings of Scheller and Raupp^39^, and in order to apply a realistic concentration, these were applied at a rate of 20 µg N g^−1^ dry soil. Hydroxylamine and NH_4_^+^ were applied at equal N rates for comparative purposes.

According to Anderson and Domsch^61^ cycloheximide, a fungal inhibitor, was applied at a rate of 8 mg g^−1^ soil and streptomycin, a bacterial inhibitor, at a rate of 5 mg g^−1^ soil. Both chemicals were applied as a dry powder on to the soil surface and subsequently mixed into the soil with a spatula for 1 min. The combined inhibition included the simultaneous application of cycloheximide and streptomycin and was designed to inhibit both bacteria and fungi. Sterilizing (as complete microbial inhibition) was performed by heating the soil. This was achieved by microwaving the soil in the jars for 4 minutes, remoistening the dry soil, and then microwaving the jars for another 3 minutes, as microwave heating is a proven method to stop microbial activities^62,63^. Thereafter, the microwaved soils were readjusted to 50% water-holding-capacity and also mixed for 1 minute. The control treatment contained urea, but no inhibitors were applied, and the soil was mixed to replicate the physical disturbance of the other treatments. Immediately after application of the inhibitor treatments the N substrate treatments were applied according to treatment at a rate of 20 µg N g^−1^ dry soil, without subsequent mixing.

In addition, three further control treatments were set up; a positive control (soil with urea but no N substrate or inhibitor addition (n = 3), also physically mixed on Day 8; a negative control (n = 3) consisting of soil without urea, inhibitors, or N substrates, also physically mixed on Day 8; and a separate NO_2_^−^ control (soil with urea but no N substrate addition, physically mixed on Day 8) for soil NO_2_^−^-N sampling at 4 different times over the duration of the experiment.

### Gas sampling and analysis

On Day −2, −1, 0, 1, 2, 4, 6, 7, 8 (before inhibitor application), 9, 10 and 11, the jars were sealed with lids equipped with rubber septa. Jar headspace gas samples were taken with a plastic syringe, fitted with a three-way-stop cock and a 25G hypodermic needle, and injected into a previously evacuated Exetainer® vials (Labco Ltd., High Wycombe, UK). The first gas sample (12 mL) was taken immediately after sealing the jar headspace. The second gas sample was taken after 1 h, only from the positive control to verify the linearity of the increase in the headspace gas concentration, and the third gas sample was taken after a 2 h incubation time (12 mL, all jars). On Days 8, 9, 10 and 11, the third gas sample (30 mL), was split between a 6 mL Exetainer® that received 12 mL, and an evacuated and helium flushed 12 mL Exetainer® that received 18 mL for ^15^N-N_2_O determination.

Nitrous oxide concentrations were determined using a gas chromatograph (SRI-8610, SRI Instruments, Torrance, CA) coupled to an autosampler (Gilson 222XL; Gilson, Middleton, WI) equipped with a ^63^Ni electron capture detector^64^. PeakSimple 4.44 software (SRI Instruments, Torrance, CA) and several N_2_O standards (range 0–100 µL L^−1^,BOC, New Zealand) were used to determine the N_2_O concentrations. The N_2_O fluxes (µg N_2_O-N m^−2^ h^−1^) were determined using the following equation:

$${N}_{2}O\,flux=(\frac{V\times \Delta {N}_{2}O\times P}{R\times T})\times {m}_{N}\times {t}^{-1}\times {A}^{-1}$$

V = headspace volume (L). ΔN_2_O = change in headspace N_2_O concentration during sampling (µL L^−1^). P = pressure (atm). R = gas constant (0.08206 L atm K^−1^ mol^−1^). T = temperature (K). m_N_ = mass of N per mole of N2O (g mol^−1^). t = time (h). A = soil surface area (m^2^).

The ^15^N enrichment of the N_2_O evolved was determined by analysing the gas samples with a continuous-flow-isotope ratio mass spectrometry CFIRMS (Sercon 20/20; Sercon, Chesire, UK) inter-faced with a TGII cryofocusing unit (Sercon, Chesire, UK). If required, gas samples were diluted by injecting 4 mL of sample gas into a helium-filled 12 mL Exetainer® (1:4 dilution).

The measured ^15^N concentration of the headspace N_2_ was close to natural abundance thus a determination of the N_2_ flux was not possible, hence, the N_2_ emissions were not considered further.

### Codenitrification calculations

As previously reported^20^ conventional denitrification produces N_2_O (non-hybrid N_2_O) while N_2_O produced via codenitrification results in an N atom from NO_2_^−^ and an N atom from a co-metabolised compound producing a hybrid N-N species, such as N_2_O. The following calculations determine the codenitrification flux, assuming that hybrid N_2_O only arises from codenitrification. We do not distinguish between the roles of biotic and abiotic reactions in this process. However, the use of biological inhibitors and soil sterilization indicate the relative roles of abiotic and biotic processes in producing hybrid N_2_O.

For the N_2_O evolved it was assumed that this was generated from one ^15^N enriched pool-fraction (d′_D_) with ^15^N enriched N (^15^N atom fraction q′_D_), and a fraction (d′_N_, equal to 1 − d′_D_) derived from a pool or pools at natural abundance (^15^N atom fraction q′_N_).

The ratios r’_1_ and r’_2_, were determined from the N_2_O m/z ion currents at m/z 44, 45 and 46^65^:

1 $${r^{\prime} }_{1}={}^{45}i/{}^{44}i$$

2 $${r^{\prime} }_{2}={}^{46}i/{}^{44}i$$

where, ^44^*i*, ^45^*i* and ^46^*i* represent the ion-currents of the N_2_O mass fractions 44, 45 and 46.

Then, following Arah^65^ (equations 22 and 23), the values of the ^15^N atom fraction of the sample (*a*′_*s*_) and the ^46^N_2_O component of the molecular fraction, of the N_2_O molecule, in the sample (*x*′_*s*_) were calculated using *r*′_1_ and *r*′_2_, while allowing for the presence of oxygen isotopes.

In Arah^65^ a′_s_ and x′_s_ are defined as follows:

3 $${{\rm{a}}^{\prime} }_{{\rm{s}}}=(1-{{\rm{d}}^{\prime} }_{{\rm{D}}}-{{\rm{d}}^{\prime} }_{{\rm{N}}})\ast {{\rm{a}}^{\prime} }_{{\rm{A}}}+{{\rm{d}}^{\prime} }_{{\rm{D}}}\ast {{\rm{a}}^{\prime} }_{{\rm{D}}}+{{\rm{d}}^{\prime} }_{{\rm{N}}}\ast {{\rm{a}}^{\prime} }_{{\rm{N}}}$$

4 $${{\rm{x}}^{\prime} }_{s}=(1-{{\rm{d}}^{\prime} }_{{\rm{D}}}-{{\rm{d}}^{\prime} }_{{\rm{N}}})\ast {{{\rm{a}}}^{2^{\prime} }}_{{\rm{A}}}+{{\rm{d}}^{\prime} }_{{\rm{D}}}\ast {{{\rm{a}}}^{2^{\prime} }}_{{\rm{D}}}+{{\rm{d}}^{\prime} }_{{\rm{N}}}\ast {{{\rm{a}}}^{2^{\prime} }}_{{\rm{N}}}$$

When letting d′_N_ equal (1 − d′_D_) and a′_A_ equal the ^15^N enrichment at natural abundance (0.003663) Eqs 3 and 4, when set to equal zero, become:

5 $$0={{\rm{d}}^{\prime} }_{{\rm{D}}}\ast {{\rm{a}}^{\prime} }_{{\rm{D}}}+(1-{{\rm{d}}^{\prime} }_{{\rm{D}}})\ast 0.003663-{{\rm{a}}^{\prime} }_{{\rm{s}}}$$

6 $$0={{\rm{d}}^{\prime} }_{{\rm{D}}}\ast {{{\rm{a}}}^{{\rm{2}}^{\prime} }}_{{\rm{D}}}+(1-{{\rm{d}}^{\prime} }_{{\rm{D}}})\ast {0.003663}^{2}-{{\rm{x}}^{\prime} }_{s}$$

Since a′_s_ and x′_s_ are known the values of d′_D_ and a′_D_ can be determined using the Solver function in Microsoft Excel^TM^, while setting the target value at zero, with the result accepted when the target value is <1 × 10^−5^.

Then the codenitrification flux was calculated according to Clough *et al*. (2001) as:

7 $${{\rm{d}}}_{{\rm{CD}}}=-\,\Delta {}^{45}{\rm{R}}{p}_{1}^{2}/(\,-\,{\Delta }^{45}{\rm{R}}{p}_{1}^{2}+\Delta {}^{45}{\rm{R}}{p}_{1}{p}_{2}+{q}_{1}{p}_{2}-{q}_{2}{p}_{1})$$

were d_CD_ is the fraction of N_2_O within the headspace derived from codenitrification and Δ^45^R is the ^45^N_2_O/^44^N_2_O ratio, while *p*_1_ (0.9963) and *q*_1_ (0.0037) are fractions of ^14^N and ^15^N in the natural abundance pool, and where *q*_2_ equals *a*’*D*, derived above, with *p*_2_ equal to 1 − *q*_2_.

Finally the codenitrification flux was determined as:

8 $${{\rm{N}}}_{2}{{\rm{O}}}_{{\rm{CD}}}={{\rm{d}}}_{{\rm{CD}}}\times ({\rm{total}}\,{{\rm{N}}}_{2}{\rm{O}}\,{\rm{flux}})$$

### Surface pH and inorganic-N measurement

Surface pH was measured on Days −2, 0, 1, 3, 5, 7, 9 and 11, by adding one drop of deionised water to the soil surface and then placing a flat surface pH probe (Broadley James Corp., Irvine, California) onto the soil surface.

The NO_2_^−^ concentration in the unmixed NO_2_^−^ control (soil + urea solution) was determined by subsampling soil with a corer (diameter 1.6 cm, depth 1.5 cm). The soil was then blended with 2 M potassium chloride (KCl), adjusted to pH 8 with potassium hydroxide^66^ at a 1:6 ratio. This procedure was performed on Days 1, 4, 6 and 10.

Subsamples of moist soil (4 g dry weight) were taken after Day 11, from the positive and negative controls, and extracted with 2 M KCl in order to determine the NH_4_^+^ and NO_3_^−^ concentrations at the end of the experiment^67,68^. Inorganic-N concentrations in the extracts were determined using Flow Injection Analysis^67^.

### Statistics

The single jars were defined as experimental units by the independent applications of treatments. The experiment focussed on achieving the most sensitive test of treatment differences and inference is not claimed for a population wider than the paddock, used for sampling. All statistical analyses were performed using SigmaPlot 13.0 (Systat Software Inc., Chicago). For each variable of interest a general linear model (ANOVA equivalent) was fitted with N substrate treatment or a factorial combination of N substrate treatment and inhibition method as explanatory variables. Using this method, the different inhibition treatments within each N substrate treatment were compared. Tests for normality (Shapiro-Wilk test) and variance (Brown-Forsythe test) were used to evaluate the residuals and define the most powerful test for each comparison of means. Hence, means comparisons were adjusted for multiplicity using Tukey, Holm-Sidak, Dunn’s or Student’s t-test adjustments to *p* values.
